# Role of spindle assembly checkpoint proteins in gametogenesis and embryogenesis

**DOI:** 10.3389/fcell.2024.1491394

**Published:** 2025-01-22

**Authors:** Renju Pun, Brian J. North

**Affiliations:** Biomedical Sciences Department, School of Medicine, Creighton University, Omaha, NE, United States

**Keywords:** spindle assembly checkpoint, embryogenesis, gametogenesis, mitotic checkpoint, mitosis

## Abstract

The spindle assembly checkpoint (SAC) is a surveillance mechanism that prevents uneven segregation of sister chromatids between daughter cells during anaphase. This essential regulatory checkpoint prevents aneuploidy which can lead to various congenital defects observed in newborns. Many studies have been carried out to elucidate the role of proteins involved in the SAC as well as the function of the checkpoint during gametogenesis and embryogenesis. In this review, we discuss the role of SAC proteins in regulating both meiotic and mitotic cell division along with several factors that influence the SAC strength in various species. Finally, we outline the role of SAC proteins and the consequences of their absence or insufficiency on proper gametogenesis and embryogenesis *in vivo*.

## 1 Introduction

Cell division is a critical feature of embryonic development (Kumar et al., 2015). Fertilization of a secondary oocyte, a haploid cell arrested in metaphase II of meiosis, by a spermatozoon results in a diploid zygote, which then divides into 2 cells through the process of mitosis (Yeste et al., 2017). This is followed by extensive cell division, which combined with various cellular signaling and morphogenic events promotes embryogenesis including blastulation, gastrulation, neurulation, and organ development (Zhai et al., 2022). Cell division during embryonic development must be carefully monitored since error prone division produces aneuploidy that has severe consequences for fecundity (Jia et al., 2015). The eukaryotic cell cycle consists of interphase (comprising the G1, S, and G2 phases) and mitosis, with each phase orchestrated by regulatory checkpoints mediated by cyclins and cyclin-dependent kinases (CDKs) (Wang, 2021). During the G1 phase, the restriction checkpoint ensures the cell is prepared to enter the cell cycle by ensuring any DNA damage is properly repaired and the cell is receiving the appropriate growth signals before committing to division (Bertoli et al., 2013). Cyclin D-CDK4/6 complexes drive progression through G1 by phosphorylating the retinoblastoma (Rb) protein, releasing E2F transcription factors to activate genes necessary for the transition into S phase and carrying out DNA synthesis (Bertoli et al., 2013; Topacio et al., 2019). During the G1/S phase transition, Cyclin E-CDK2 initiates DNA replication by activating origins of replication. During S phase, Cyclin A replaces Cyclin E, terminating DNA synthesis and facilitating the transition into the G2 phase (Ding et al., 2020). The G2 phase is regulated by the G2/M checkpoint, which ensures the integrity of replicated DNA before mitotic entry (Krempler et al., 2007; Kousholt et al., 2012). Cyclin A-CDK1 activity orchestrates the preparatory events for mitosis by promoting the nuclear translocation of Cyclin B1-CDK1 (Gong et al., 2007; Gong and Ferrell, 2010). Once in the nucleus, Cyclin A and Cyclin B1-CDK1 collaborate to drive key mitotic processes, including chromosome condensation and nuclear envelope breakdown (Petrone et al., 2016; Enserink and Kolodner, 2010; Gong and Ferrell, 2010). To ensure fidelity in chromosome segregation and prevent aneuploidy, the spindle assembly checkpoint (SAC) operates during M phase, monitoring the attachment of chromosomes to the spindle apparatus to guarantee proper segregation between daughter cells (Lara-Gonzalez et al., 2012; Lara-Gonzalez et al., 2021b). A variety of studies have been published over the last decade that have produced intriguing insights into the role of the SAC, and the factors that regulate SAC function, in gametogenesis and embryogenesis. In this review, we will explore the role of SAC proteins during these crucial biological processes.

## 2 The spindle assembly checkpoint (SAC)

Segregation of genetic material between daughter cells begins during the anaphase stage of mitosis (Musacchio and Desai, 2017). However, the mechanisms that ensure each daughter cell receives an equal and full complement of DNA, begin earlier in the cell division process. During S phase, cells fully duplicate each chromosome (Musacchio and Desai, 2017). Additionally, centrosome duplication also occurs during S phase (Tsou and Stearns, 2006; Holland et al., 2010) and errors in centrosome duplication can cause chromosome missegregation leading to aneuploid daughter cells (Silkworth et al., 2009; Ganem et al., 2009). During prometaphase of mitosis, a complex of proteins called the kinetochore assembles at the centromere of each chromosome, serving as the attachment site for microtubules from the mitotic spindle (Musacchio and Desai, 2017). Kinetochore associated proteins regulate the movement of each sister chromatid in opposing directions along spindle fiber microtubules during anaphase (Musacchio and Desai, 2017). Therefore, it is imperative for microtubules from the bipolar spindle to attach to kinetochores of both chromatids (amphitelic attachment) for the divergent movement and even distribution of chromosomes to each daughter cell (Lampson and Grishchuk, 2017). However, various challenges can compromise the fidelity of chromosome segregation, such as syntelic attachment where both sister chromatids connect to the same spindle pole, merotelic attachment in which a single chromatid or kinetochore attaches to both spindle poles, or monotelic attachment in which only one sister chromatid is attached to spindle microtubules (Lampson and Grishchuk, 2017; Khodjakov and Pines, 2010; Taylor et al., 2004). If these issues are not resolved, they can lead to chromosome missegregation (Lampson and Grishchuk, 2017). While the SAC monitors unattached kinetochores, it does not reliably detect all attachment errors, such as merotelic and syntelic attachments (Cimini et al., 2001; Jin et al., 2012). Loss or gain of chromosomes, termed aneuploidy, results in severe consequences such as developmental defects and cancer (Shahbazi et al., 2020; Boveri, 2008). Trisomy 21 is one such instance in which an extra copy of chromosome 21 is acquired during abnormal cell division leading to Down’s syndrome (Roizen and Patterson, 2003). Therefore, each sister chromatid must bi-orient and attach to microtubules during metaphase before anaphase initiates. The SAC, also known as the mitotic checkpoint, fulfills this need as a surveillance mechanism to confirm all chromosomes are properly aligned and bound by bipolar spindles to ensure anaphase fidelity (Lara-Gonzalez et al., 2012).

### 2.1 Activation of the SAC

The SAC prevents the progression to anaphase until all chromosomes are properly attached to the mitotic spindle via their kinetochores (Musacchio, 2015b; London and Biggins, 2014; Cheeseman, 2014). The SAC is activated by unattached or improperly attached kinetochores, which generate a ‘wait’ signal that halts anaphase progression. This signal prevents the activation of the anaphase-promoting complex/cyclosome (APC/C), ensuring that cells do not prematurely segregate their chromosomes (McAinsh and Kops, 2023). A multitude of studies have been carried out to decipher what exactly the SAC senses leading to its activation (McVey et al., 2021; Khodjakov and Pines, 2010). Traditionally, it is believed that the SAC acts as a quality control mechanism, preventing the cell from segregating its chromosomes (progressing from metaphase to anaphase) if there are errors in chromosome attachment to the bipolar spindle (Rieder et al., 1995). According to this view, the SAC is primarily concerned with whether the chromosomes are correctly attached to the spindle microtubules. This suggests that the SAC is unable to monitor for proper orientation of the chromatids. For instance, chromatids can be mono-oriented or bi-oriented regardless of proper kinetochore attachment (amphitelic) (Khodjakov and Pines, 2010). This view is supported by studies using laser beam-mediated ablation of unattached kinetochore on a monotelic chromosome that resulted in a normal progression of mitosis suggesting that the SAC only detects the presence or absence of microtubule attachment on the kinetochores (Rieder et al., 1995). Another mechanistic explanation behind SAC activation that has been extensively studied is based on the lack of tension across unattached kinetochores (Mukherjee et al., 2019; Chen et al., 2021). For instance, applying tension to an unattached kinetochore through a micromanipulation needle releases cells from metaphase and they progress into anaphase suggesting that tension between kinetochores of sister chromatids regulates SAC activation (Li and Nicklas, 1995; Nicklas and Koch, 1969). Moreover, there is a correlation between the level of tension and metaphase delay time suggesting that the cell is highly sensitive to tension levels, which may serve as a critical signal to prevent chromosome segregation errors during mitosis (Mukherjee et al., 2019). Hence, the SAC is thought to sense either the presence of unattached kinetochores or the lack of tension across the kinetochores of sister chromatids. Although strong evidence exist for both explanations, the ability of the kinase Aurora B to contribute to both mechanisms may connect these mechanisms together (McVey et al., 2021). Aurora B has been proposed to sense the lack of tension in the kinetochores and translate this into a biochemical signal by phosphorylating members of the kinetochore such as Knl1 and Mis12 that belong to the KMN network (Akiyoshi et al., 2010; Welburn et al., 2010; Cheeseman et al., 2002). Another study suggests that Aurora B activity is not directly regulated by tension (Liu et al., 2009). Instead, phosphorylation depends on the spatial proximity of substrates to Aurora B at the inner centromere. This proximity or distance, is affected by tension which indirectly affects the phosphorylation of the substrates by Aurora B (Liu et al., 2009). Such a spatial model underscores a nuanced mechanism by which Aurora B may modulate kinetochore signaling.

The KMN network is a large assembly of proteins composed of three main subcomplexes, namely, the Mis12 complex, the Ndc80 complex, and the Knl1 complex (Varma and Salmon, 2012). The Mis12 complex, consisting of four proteins (Dsn1, Mis12, Nnf1, and Nsl1), is responsible for the assembly of the Ndc80 and Knl1 complexes along the outer kinetochore and has been described as a protein interaction hub for outer kinetochore assembly (Cheeseman and Desai, 2008; Maskell et al., 2010; Petrovic et al., 2010). The Knl1 and Ndc80 complexes are tethered by Nsl1 aiding in their recruitment to the kinetochore (Petrovic et al., 2010). The Ndc80 complex has four subunits (Hec1, Nuf2, Spc24, and Spc25) in a coiled coil fashion and is responsible for bridging the KMN network to microtubules (Cheeseman and Desai, 2008; Ciferri et al., 2008; Wei et al., 2007). The Ndc80 complex provides a docking site for the N-terminal region of the SAC protein Mps1 (Kemmler et al., 2009; Nijenhuis et al., 2013). Studies have shown that the Mps1 and microtubules compete against each other to bind to Ndc80 (Pleuger et al., 2024). According to this competition model, Mps1 directly interacts with the HEC1 domain of the NDC80 complex and initiates the SAC (Pleuger et al., 2024; Ji et al., 2015). However, the presence of end-on microtubule attachment to the NDC80 complex disrupts this interaction (Pleuger et al., 2024). This competitive binding mechanism serves as a direct sensor for kinetochore-microtubule attachment status, regulating SAC signaling (Ji et al., 2015; Pleuger et al., 2024). However, recent findings challenge this direct competition model showing that Mps1 autophosphorylation is responsible for its release from kinetochores at least in the context of native kinetochores isolated from yeast (Koch et al., 2019). These findings suggest that while direct competition between Mps1 and microtubules for Ndc80 binding may contribute to SAC regulation, Mps1 autophosphorylation likely plays a key role in modulating its kinetochore association.

**FIGURE 1:**
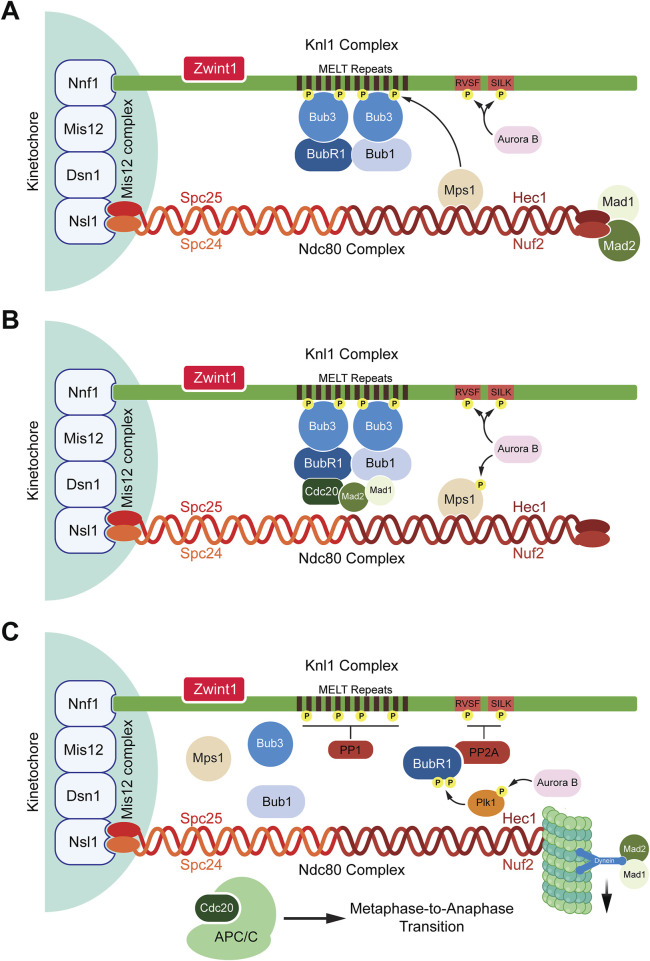
Spindle assembly checkpoint activation and deactivation. **(A)** SAC activation: When kinetochores are unattached to microtubules, the SAC is activated. Aurora B recruits Mps1 that binds to the Ndc80 complex and phosphorylates the MELT repeats present on the Knl1 complex. Once phosphorylated, the MELT repeats recruit the SAC protein Bub3 which further recruits BubR1 and Bub1. Aurora B also phosphorylates the RVSF and SILK domains preventing the phosphatase PP1 from binding to Knl1 complex. **(B)** SAC maintenance: Bub1 interacts with Cdc20 and the Mad1:Mad2 complex bringing Mad2 and Cdc20 in close proximity to form a subcomplex inhibiting the interactions between APC/C and Cdc20. The assembled MCC now consists of the Bub3:BubR1 and Mad2:Cdc20 subcomplexes. **(C)** SAC deactivation: Once microtubules attach to the Ndc80 complex, Mps1 is displaced from the Ndc80 complex, Aurora B activates Plk1 which stimulates the binding of PP2A to BubR1 by phosphorylating BubR1. PP2A dephosphorylates the RVSF and SILK domains allowing for PP1 to bind to Knl1 where it dephosphorylates the MELT repeats. This causes the release of MCC components which are stripped away from the Knl1 complex by dynein proteins. Hence, the SAC is deactivated and the binding of APC/C to Cdc20 occurs to allow for the metaphase-to-anaphase transition.

Once docked on Ndc80, Mps1 phosphorylates Knl1 which is one of the two subunits of the Knl1 complex, the other being Zwint1. Phosphorylation of Knl1 occurs on threonine residues present on its MELT repeats, triggering Knl1 to act as a docking site for SAC proteins BubR1, Bub1, and Bub3 (London et al., 2012; Shepperd et al., 2012; Yamagishi et al., 2012; Krenn et al., 2014; Overlack et al., 2015; Vleugel et al., 2013; Zhang et al., 2014). The dephosphorylation of Knl1 is prevented by Aurora B which phosphorylates the SILK and RVSF motifs at the N-terminus of Knl1 (Agarwal and Varma, 2015). These phosphorylation events prevent protein phosphatase 1 (PP1) from binding to Knl1. It has also been speculated that Aurora B promotes the recruitment of Mps1 to the KMN network (Figure 1) (Agarwal and Varma, 2015). The coordinated assembly and regulation of the KMN network, particularly through the actions of key protein complexes such as Mis12, Ndc80, and Knl1, are crucial for the proper activation of the SAC (Agarwal and Varma, 2015). These interactions ensure accurate chromosome segregation by tightly controlling kinetochore-microtubule attachments and signaling pathways, highlighting the intricate molecular mechanisms that maintain genomic stability during mitosis (Figure 1A).

### 2.2 The mitotic checkpoint complex (MCC)

The SAC ensures accurate chromosome segregation by preventing anaphase onset until all kinetochores are properly attached to spindle microtubules and tension is generated across sister chromatids. Upon detecting unattached kinetochores, the SAC activates and assembles a complex of proteins collectively known as the mitotic checkpoint complex (MCC) (Lara-Gonzalez et al., 2012; Jia et al., 2013; Musacchio, 2015b; Musacchio and Desai, 2017). The MCC consists of four proteins, Mad2, BubR1, Bub3, and Cdc20 (Chao et al., 2012; Hardwick et al., 2000; Sudakin et al., 2001; Tang et al., 2001). The primary function of the MCC is to inhibit the activation of the APC/C, an E3 ubiquitin ligase responsible for initiating anaphase (Lara-Gonzalez et al., 2021b; Barford, 2020). Several proteins aid in the assembly of the MCC complex such as Bub1, Mad1, and Mps1 (London and Biggins, 2014; Fischer et al., 2021; Kulukian et al., 2009; Hewitt et al., 2010; Abrieu et al., 2001). The process begins with Mps1 kinase phosphorylating the MELT repeats of the kinetochore protein Knl1 followed by recruitment of Bub1 and Bub3 to the kinetochores (London et al., 2012; Shepperd et al., 2012; Yamagishi et al., 2012). Bub1-Bub3 binding increases the affinity of Bub3 towards the phosphorylated MELT repeats (Primorac et al., 2013). Bub3 also interacts with BubR1 which leads to the formation of a heterotetrametric complex containing Bub1-Bub3 and BubR1-Bub3 dimers present on the kinetochores (Overlack et al., 2015; Zhang et al., 2015). While BubR1 also interacts with Bub1, studies have shown that the recruitment of BubR1 to the kinetochores by Bub1 is dispensable for SAC activation (Vleugel et al., 2015). The recruitment of the other two MCC proteins, Mad2 and Cdc20, occurs through the interaction of multiple proteins (Ge et al., 2009). Bub1 recruits and stabilizes the Mad1-Mad2 complex at unattached kinetochores. This occurs via Bub1s CM1 motif, which binds the RLK motif of Mad1 (London and Biggins, 2014; Zhang et al., 2017; Lara-Gonzalez et al., 2021b; Klebig et al., 2009). Simultaneously, ABBA motif of Bub1 interacts with the WD40 domain of Cdc20 (Piano et al., 2021; Lara-Gonzalez et al., 2021b). These events bring Mad2 and Cdc20 in close proximity (Piano et al., 2021; Lara-Gonzalez et al., 2021a). Mad1, when phosphorylated by Mps1 on its RWD domain, also interacts with Cdc20 which exposes the Mad2 binding motif present within the Cdc20 N-terminus (Faesen et al., 2017; Ji et al., 2017; Lara-Gonzalez et al., 2021a; Piano et al., 2021). BubR1 also interacts with Cdc20 through its KEN box (K1), ABBA motif, and D box (D2) domains (Burton and Solomon, 2007; King et al., 2007; Lara-Gonzalez et al., 2011). Together, these events bring Mad2, BubR1, Bub3, and Cdc20 into close proximity, culminating in the formation of a complete MCC complex (Figure 1B).

A primary function of the MCC is to inhibit APC/C activation by sequestering its coactivator, Cdc20. Without APC/C activity, key substrates such as Securin and Cyclin B1 remain stabilized (Barford, 2020). The APC/C ubiquitinates Securin and Cyclin B1, marking them for proteasome-dependent degradation (Barford, 2020). Securin inhibits Separase, an enzyme required for cleaving the cohesin subunit RAD21, which holds sister chromatids together (Anderson et al., 2002; Haering et al., 2002). Cyclin B1, on the other hand, is the regulatory subunit of the mitotic kinase CDK1 which is critical for the G2 to M phase transition (Zur and Brandeis, 2002). During anaphase, APC/C-mediated ubiquitination of Cyclin B1 marks it for degradation, leading to the inactivation of CDK1 (Zur and Brandeis, 2002). This inactivation is crucial for reversing key mitotic processes including nuclear envelope breakdown (NEB) and chromosome condensation (Malumbres, 2014; Qian et al., 2015). Thus, inhibition of the APC/C by the MCC prevents the degradation of its substrates, Securin and Cyclin B1, until the SAC is satisfied, ensuring proper chromosome alignment and attachment to bipolar spindles prior to anaphase onset.

### 2.3 The MCC:APC/C:Cdc20 complex

It has long been accepted that the primary function of the MCC is to prevent the formation of the APC/C^Cdc20^ complex by binding to Cdc20 (Sudakin et al., 2001). This implies that the MCC competes with the APC/C for available Cdc20. Recent observations have forced revisions to this model and have provided an elegant explanation for how the MCC inhibits the activity of APC/C (Herzog et al., 2009; Alfieri et al., 2016; Yamaguchi et al., 2016; Kelly et al., 2014). First, the MCC consists of BubR1:Bub3:Cdc20:Mad2 in a 1:1:1:1 stoichiometric ratio (Sudakin et al., 2001; Alfieri et al., 2016; Yamaguchi et al., 2016; Izawa and Pines, 2015). One Cdc20 is associated with the MCC (Cdc20_M_). It is bound to the MCC through its interaction with BubR1 by binding to the KEN box, TPR motifs, ABBA motifs, and the D box that Cdc20_M_ recognizes within BubR1 (Di Fiore et al., 2015; Diaz-Martinez et al., 2015; Lischetti et al., 2014). Cdc20_M_ also binds to the MCC through interactions with Mad2 and Mad3 (Izawa and Pines, 2015; Yamaguchi et al., 2016). Second, BubR1 consists of a second set of KEN box (K2), ABBA motifs (A1), and the D box (D1) that is recognized by another Cdc20 which is associated with the APC/C and is termed Cdc20_A_ (Alfieri et al., 2016; Yamaguchi et al., 2016). Recent studies have demonstrated a direct interaction between Cdc20_A_ and Cdc20_M_ (Zhang et al., 2024). The CRY box, an unconventional degron motif within Cdc20, plays a critical role in this interaction. Electrostatic interactions are present between the CRY box residues (R162, K163) of Cdc20_M_ and acidic residues (E180, D203) of Cdc20_A_ (Zhang et al., 2024). Mutations in the CRY box, such as R162E/K163E or E180R/D203R, disrupt these interactions, abolishing SAC function and accelerating mitosis. Additionally, the CRY box in Cdc20_M_ interacts with the KNOT domain of BubR1, consisting of a D-box pseudo-degron sequence (residues 224–232) and a hydrophobic loop, further stabilizing the MCC. Mutations that unfold the KNOT domain lead to SAC defects as well (Zhang et al., 2024). These findings suggest the functional implications of a Cdc20_A_ and Cdc20_M_ interaction for SAC function.

The interaction of Cdc20_A_ with BubR1 shifts it away from the D-box receptor, which is formed by Cdc20_A_ and APC10 within the APC/C (Izawa and Pines, 2015; Alfieri et al., 2016; Yamaguchi et al., 2016). Cdc20_A_ and APC10 together recognize APC/C substrates and target them for ubiquitination (Buschhorn et al., 2011; da Fonseca et al., 2011). Therefore, preventing the interaction of Cdc20_A_ and APC10 prevents substrate recognition. An additional mechanism by which BubR1 in the MCC prevents APC/C activity is through blocking recruitment of ubiquitination machinery. Since APC/C is an E3 ubiquitin ligase, it requires an E2 ubiquitin conjugating enzyme to add ubiquitin to substrates (Yang et al., 2021). The APC/C recruits two E2s, namely, UbcH10 (also known as UBE2C) and UBE2S, to its catalytic core composed of APC2 and APC11 (Brown et al., 2016; Brown et al., 2014). BubR1 utilizes its TPR binding motifs to interact with APC2 and sterically hinder UbcH10 from binding to the catalytic subunit on APC/C (Yamaguchi et al., 2016). Hence, the MCC functions to inhibit the APC/C via multiple mechanisms during the SAC.

### 2.4 SAC deactivation

Once bipolar microtubules attach to kinetochores of all chromosomes, the SAC must be silenced which involves multiple mechanisms. Three major processes have been characterized that silence the SAC.

1) Cdc20 ubiquitination: Since the MCC inhibits the APC/C, its disassembly is necessary to initiate anaphase. Specifically, Cdc20 that is a part of the MCC, Cdc20_M,_ is ubiquitinated by the APC/C itself (Nilsson et al., 2008). Recent studies have clarified how Cdc20_M_ gets ubiquitinated by the APC/C and is preferred over Cdc20_A_ (Alfieri et al., 2016; Yamaguchi et al., 2016). The catalytic core of the APC/C consists of APC2, which contains a Cullin domain, and APC11, which has a RING domain involved in recruiting the two E2 ubiquitin-conjugating enzymes, UBE2C and UBE2S (Yamaguchi et al., 2016). UBE2C is essential for substrate ubiquitination, while UBE2S is required for ubiquitin chain elongation (Yamaguchi et al., 2016). The MCC inhibits the recruitment of these E2 enzymes by binding to APC2 and APC11. Specifically, BubR1 binds to the WHB domain of APC2 and the RING domain of APC11 (Yamaguchi et al., 2016). This represents the closed state of the APC/C, where its catalytic cavity is blocked. In its open conformation, the MCC complex shifts away from the catalytic cavity, allowing UBE2C to bind to the WHB domain of APC2 and the RING domain of APC11 (Alfieri et al., 2016; Yamaguchi et al., 2016). This positioning places Cdc20_M_ near the APC2-APC11 Cullin-RING complex, activated by UBE2C, which then ubiquitinates Cdc20_M_ (Alfieri et al., 2016; Yamaguchi et al., 2016). This is supported by the observation that deletion of APC15, another component of the APC/C, enhances the MCC:APC/C interaction and reduces Cdc20_M_ ubiquitination suggesting the presence of complexes within the APC/C that target Cdc20_M_ (Foster and Morgan, 2012). Overall, Cdc20_M_ ubiquitination releases it from the MCC (Alfieri et al., 2016; Yamaguchi et al., 2016). However, whether it is degraded or not remains unclear. This also leads to the proper interaction between Cdc20_A_ and APC10 which allows for APC/C to interact with its substrates, including Securin and Cyclin B1, for ubiquitination and subsequent degradation (Alfieri et al., 2016; Yamaguchi et al., 2016).
2) Mad2:Cdc20 disassembly: Like the MCC:APC/C:Cdc20 super complex, Mad2 exists in either an open or closed conformation (Habu et al., 2002). In its closed form, Mad2 interacts with Mad1 and Cdc20 to participate in the MCC (Habu et al., 2002). Therefore, it is vital to reestablish Mad2 in its open conformation to silence the SAC. TRIP13, an AAA-ATPase, uses an adaptor protein termed p31^comet^ to promote the conversion of the closed Mad2 to an open conformation (Wang et al., 2014; Eytan et al., 2014; Ye et al., 2015; Musacchio, 2015a). Interestingly, the reestablished open form of Mad2 is also essential for SAC activation since TRIP13-deficient cells, which contain only the closed form of Mad2, cannot undergo SAC activation (Ma and Poon, 2018).
3) Phosphatases: Phosphorylation plays a key role in the initiation of the SAC. The phosphorylation of Knl1 at the MELT repeats by Mps1 initiates SAC signaling (Zhang et al., 2014). Knl1 is also phosphorylated at the SILK and RVSF motifs by Aurora B which abrogates the recruitment of phosphatases preventing SAC silencing (Nasa et al., 2018; Caldas and DeLuca, 2014). Once kinetochores are attached to microtubules on Ndc80, this dislodges Mps1 from kinetochores (Ji et al., 2015; Ji et al., 2017). While phosphorylation events initiate SAC assembly, dephosphorylation is crucial for SAC silencing. Two phosphatases, PP2A and PP1, are key players in this process (Foley et al., 2011; Liu et al., 2010; Posch et al., 2010). Plk1 triggers the binding of PP2A to BubR1 by further phosphorylating Serine-676 and Threonine-680 residues present on the kinetochore attachment regulatory domain (KARD) motif of BubR1 (Elowe et al., 2007; Suijkerbuijk et al., 2012; Kruse et al., 2013; Wang et al., 2016a; Wang et al., 2016b). The regulatory subunit of PP2A, B56, binds to BubR1 on its phosphorylated KARD domain (Suijkerbuijk et al., 2012; Ghongane et al., 2014). PP2A then dephosphorylates the SILK and RVSF motifs of Knl1 (Espert et al., 2014). This dephosphorylation counters the kinase activity of Aurora B allowing access for the phosphatase PP1 to dock on Knl1 and dephosphorylate the MELT repeats (Nijenhuis et al., 2014). Recent studies have further elucidated the role of PP2A/B56 in dephosphorylation processes critical for SAC signaling. PP2A/B56 directly targets the MELT repeats of Knl1, as demonstrated by the dephosphorylation of Knl1 at Threonine-875, a conserved Mps1 phosphorylation site (Espert et al., 2014). Loss of PP2A/B56 activity results in increased Threonine-875 phosphorylation (Espert et al., 2014). Additionally, PP2A/B56 bound to BubR1 can dephosphorylate Bub1 at Threonine-461 (Wang et al., 2023). This residue is phosphorylated by Mps1 creating a binding site for Mad1 on Bub1 which is crucial for SAC signaling (Zhang et al., 2017). Therefore, dephosphorylation events by these phosphatases initiate SAC silencing leading to the disassembly of the MCC from the kinetochore. In order to remove the MCC proteins, the microtubule motor dynein recognizes Mad1–Mad2 at the kinetochore as cargoes and transports them away from the kinetochores towards the spindle poles along microtubules (Gassmann et al., 2010; Howell et al., 2001; Ide et al., 2023; Buffin et al., 2005). Although Bub1, BubR1, and Bub3 have been previously confirmed as dynein cargoes, a recent study suggests that SAC proteins maybe evicted from kinetochores due to dephosphorylation of kinases such as Mps1 rather than through motor activity of dynein (Ide et al., 2023) (Figure 1C). Overall, SAC signaling is a complex process involving multiple kinases and phosphatases tightly regulated to ensure an even separation of sister chromatids during mitosis.

## 3 SAC in gametogenesis

Gametogenesis is defined as the process by which mature haploid gametes develop from precursor cells known as primordial germ cells. This process involves a series of mitotic and meiotic cell divisions, followed by cell differentiation to form gametes (Larose et al., 2019). Gametogenesis occurs in the testes in males and is termed spermatogenesis during which spermatozoa are produced while females undergo oogenesis in the ovaries to produce oocytes (Larose et al., 2019). It has been observed that defective meiotic cell division during gametogenesis can lead to aneuploid embryos and embryonic lethality (Syrjanen et al., 2014). For instance, female mice lacking synaptonemal complex protein 3 (Sycp3) have aneuploid oocytes due to meiotic non-disjunction events (Syrjanen et al., 2014). The synaptonemal complex is active during prophase of meiosis-I during which homologous chromosomes are brought into proximity for synapsis and homologous recombination (Kouznetsova et al., 2005; Westergaard and von Wettstein, 1972). Deletion of Sycp3 in mice triggers a sexually dimorphic phenotype where males are infertile while the females are sub fertile and produce a high number of aneuploid secondary oocytes (Yuan et al., 2002). Despite a similar rate of fertilization, the Sycp3 null oocytes display a significantly higher level of embryonic lethality (Lightfoot et al., 2006). While these chromosomally abnormal embryos are able to implant into the uterine lining and undergo gastrulation, they fail to develop beyond E8.0. Cytological analysis show that about 57% of E3.5 blastocyst cells derived from Sycp3 null females are aneuploid with 91% of those displaying mosaic aneuploid karyotypes (Yuan et al., 2002). Histological analysis of the E7.0 aneuploid embryos have pycnotic bodies in the ectodermal layer. Pycnotic bodies show as darkly stained nuclei due to nuclear condensation during cell death (Magrassi and Graziadei, 1995). At E8.0, aneuploid embryos are severely disordered and have higher levels of pycnotic bodies (Yuan et al., 2002). TUNEL staining show that aneuploid embryos are eliminated through apoptosis. However, loss of p53 does not rescue embryonic lethality in Sycp3 null females suggesting that apoptosis occurs through a p53-independent mechanism (Lightfoot et al., 2006; Yuan et al., 2002). Furthermore, apoptosis is not due to unfaithful chromosomal segregation during mitosis as both wild-type and Sycp3 null embryos contain an equal number of cells at different stages of mitosis suggesting that mitosis is unaffected in the mosaic aneuploid cells (Yuan et al., 2002). Therefore, faithful chromosomal segregation is integral to meiosis.

The spindle assembly checkpoint is active during meiosis in mouse oocytes as demonstrated by microtubule disruption studies using nocodazole (Eichenlaub-Ritter and Boll, 1989). Treatment of metaphase I oocytes with nocodazole causes microtubule depolymerization, leading to spindle disassembly and loss of equatorial chromosome alignment. Upon recovery from nocodazole treatment, oocytes proceed successfully through meiosis I, indicating that SAC activation delays anaphase onset in response to spindle disruption (Eichenlaub-Ritter and Boll, 1989). Further support for SAC functionality comes from studies examining the SAC protein Bub1. In mouse oocytes, Bub1 localizes to kinetochores and undergoes phosphorylation during anaphase I and II (Brunet et al., 2003). This phosphorylation is crucial for SAC activity and is consistent with observations in *Xenopus* oocytes, where Bub1 is also phosphorylated and localized to kinetochores (Schwab et al., 2001). However, in contrast to Bub1 in *Xenopus* oocytes, Bub1 in mouse oocytes is not dependent on the MAPK-Rsk pathway (Schwab et al., 2001). The MAPK pathway has been previously implicated in SAC regulation, as its activity is required for nocodazole-induced metaphase arrest in *Xenopus* egg extracts (Minshull et al., 1994; Takenaka et al., 1997; Wang et al., 1997). In mammalian cells, active MAPK localizes to spindle poles and kinetochores during mitosis (Zecevic et al., 1998). In *Xenopus* oocytes, MAPK-Rsk directly phosphorylates Bub1, enabling its kinetochore localization and interaction with other SAC proteins, which is essential for checkpoint activation and metaphase arrest (Schwab et al., 2001). Furthermore, Rsk activation alone has been shown to directly phosphorylate Bub1 both *in vitro* and *in vivo* in *Xenopus* oocytes (Schwab et al., 2001). Another key SAC protein, Mad2, is recruited to kinetochores during early metaphase I in mammalian oocytes (Schwab et al., 2001). Functional studies with dominant-negative Mad2 mutants reveal that disrupting Mad2 activity prevents metaphase I arrest upon nocodazole treatment, underscoring its role in SAC-mediated checkpoint enforcement (Schwab et al., 2001). Taken together, these studies demonstrate that the SAC, including key proteins such as Bub1 and Mad2, is functional during gametogenesis.

### 3.1 SAC in oocytes versus spermatocytes

Interestingly, the strength of SAC signaling appears to be stronger in male spermatocytes compared to female oocytes. In female oocytes, treatment with low concentrations of nocodazole (0.01–0.1 µM) induces spindle abnormalities and aneuploidy without significantly arresting meiosis I, suggesting weaker SAC activation (Everett and Searle, 1995). Intermediate concentrations of nocodazole (0.2–2 µM) induces a robust SAC response, resulting in meiosis I arrest in approximately 90% of oocytes (Everett and Searle, 1995). High concentrations of nocodazole (≥10 µM) completely depolymerizes microtubules, causing chromosome scattering and a total disruption of spindle integrity (Eichenlaub-Ritter and Boll, 1989). These results suggest that lower nocodazole concentrations may cause only partial destabilization or mild spindle abnormalities, without fully disassembling microtubules. Furthermore, when oocytes treated with high nocodazole treatments are allowed to recover, they reestablish the spindle apparatus and proceed through anaphase. However, this recovery process is highly error-prone and leads to a dramatic increase in aneuploidy (Eichenlaub-Ritter and Boll, 1989), suggesting a compromised or weaker SAC function in oocytes. Interestingly, there are insufficient studies carried out with respect to nocodazole effects in spermatocytes to make direct comparisons with the oocyte data described above, and future studies in this area could help to define differences between oocytes and spermatocytes with regard to SAC response.

Oocytes derived from an XO mouse model of monosomy, which lack second sex chromosome, proceed through meiosis I even in the presence of an unpaired X chromosome (LeMaire-Adkins et al., 1997). In contrast, XO spermatocytes elicit a strong meiotic arrest during metaphase I leading to apoptosis (de Boer et al., 1991; Sutcliffe et al., 1991). Similar sex differences in SAC function are observed in *Mlh1* knockout models. *Mlh1* is a mismatch repair gene essential for proper DNA replication prior to cell division in meiosis I, and mutant *Mlh1* leads to univalent chromosomes during meiosis (LeMaire-Adkins et al., 1997). Male spermatocytes that are *Mlh1*-null halt meiosis. In contrast, *Mlh-1* null female oocytes proceed through meiosis even in the presence of univalent chromosomes and initiate anaphase (Gorbsky, 2015). This suggests that mammalian oocytes require stable bipolar attachment of some, but not all, chromosomes which increases aneuploidy and errors in female oogenesis (Nagaoka et al., 2011).

However, conclusions from these observations regarding the differential strength of SAC signaling between male spermatocytes and female oocytes is challenged by another study which utilized spermatocytes from Robertsonian centric fusion heterozygous mice (Eaker et al., 2001). Robertsonian translocations involve Robertsonian (ROB) chromosomes that are acrocentric with centromeres located towards the chromosomal ends. Individuals who are heterozygous for Robertsonian chromosomes have a higher chance of producing aneuploid gametes during meiosis I due to unbalanced segregation (Yip, 2014). Spermatocytes from Robertsonian heterozygous chromosomes display an increased level of apoptosis in meiosis I suggesting that abnormal spermatocytes are eliminated (Eaker et al., 2001). Interestingly, staining for centromeric proteins CENP-E and CENP-F in Robertsonian heterozygous spermatocytes revealed an increase in fluorescence intensity at the kinetochores of lagging or malattached chromosomes (Eaker et al., 2001). CENP-E and CENP-F facilitate the attachment of spindle microtubules to the kinetochores and interact with BubR1 and Bub1 (Ciossani et al., 2018). However, this SAC signaling is not entirely infallible, as it fails to eliminate all aneuploid gametes efficiently, leading to the production of abnormal gametes (Ciossani et al., 2018). Nevertheless, all evidence points to the fact that the SAC is active during gamete formation to ensure proper meiosis although differences in SAC strength are present in oogenesis and spermatogenesis (Vogt et al., 2008). SAC proteins and their localization dynamics differ between spermatocytes and oocytes. In oocytes undergoing meiosis I, Mad2 localizes to unattached kinetochores and dissipates once proper kinetochore-microtubule attachments are formed, similar to its behavior in mitotic cells (Kallio et al., 2000). In contrast, in spermatocytes, immunofluorescence studies on rats and mice reveal that Mad2 remains localized to kinetochores throughout meiosis I (Kallio et al., 2000). Despite these differences in Mad2 dynamics, its depletion has significant consequences in both cell types. In oocytes, reduced Mad2 levels accelerate meiosis I and lead to chromosomal missegregation, whereas Mad2 insufficiency in spermatocytes results in aneuploidy (Niault et al., 2007; Lane and Jones, 2017). The benefits behind altered SAC protein dynamics in spermatocytes is yet to be made clear.

### 3.2 SAC in meiosis versus mitosis

The SAC plays a pivotal role in ensuring accurate chromosome segregation during mitosis. However, its role in meiosis is comparatively less well understood. What is evident is that SAC proteins are both present and functionally relevant in meiosis. Mammalian oocytes, for example, express key SAC components, including MAD1, MAD2, BubR1, Bub1, Bub3, Mps1, and Aurora B (Zhang et al., 2005; Homer et al., 2005; Tsurumi et al., 2004; McGuinness et al., 2009; Li et al., 2009b; Hached et al., 2011; Lane et al., 2010; Yang et al., 2010). These SAC proteins execute similar roles in meiosis as they do in mitosis. For instance, Mad2 depletion in mouse oocytes accelerates the degradation of Securin and Cyclin B1, increases aneuploidy rates, and shortens the duration of meiosis I (Homer et al., 2005). This outcome parallels observations in mitotic cells, where Mad2 depletion in HeLa cells leads to a similar reduction in mitotic duration (Meraldi et al., 2004; Michel et al., 2001). Likewise, depletion of Bub1 in oocytes speeds up meiosis I and results in chromosome segregation errors, a phenotype mirrored in HeLa cells with Bub1 depletion, which experience significant sister chromatid segregation errors (McGuinness et al., 2009; Tang et al., 2004b). Despite these functional similarities, notable differences exist between SAC function in mitosis and meiosis. One key distinction is that the SAC response is weaker or less sensitive during meiosis. This is evident from multiple studies showing that manipulations causing chromosome misalignment in oocytes often fail to halt anaphase onset (Gui and Homer, 2012; Kolano et al., 2012; Lane and Jones, 2017; Sebestova et al., 2012; Kyogoku and Kitajima, 2017). Therefore, mammalian oocytes are prone to chromosomal segregation errors (Bennabi et al., 2016). In meiosis I, homologous chromosomes pair and recombine to form bivalents, which are held together by chiasmata. Non-aligned bivalents, which fail to properly position at the metaphase plate, risk missegregation (Lane et al., 2012). However, the presence of such non-aligned bivalents often fails to maintain SAC activation in oocytes, allowing the APC/C to remain active leading to nondisjunction (Lane et al., 2012).

The reduced SAC sensitivity in meiosis I is further exemplified by studies on NuMA (nuclear mitotic apparatus protein), which anchors microtubules to spindle poles in acentrosomal oocytes. NuMA deletion disrupts spindle assembly and chromosome alignment, yet the SAC remains silent, permitting oocytes to proceed through anaphase I despite severe defects, leading to aneuploid oocytes and infertility (Kolano et al., 2012). Similarly, in meiosis II, misaligned chromosomes fail to trigger SAC activation, allowing oocytes to progress through anaphase II resulting in further aneuploidy (Mihajlovic et al., 2023). Another significant difference is the timing and duration of SAC activity. Mitosis is a relatively fast process, lasting minutes to a few hours in human somatic cells (Rieder and Maiato, 2004; Herbert et al., 2015). Consequently, SAC activation and silencing occur rapidly, ensuring efficient progression through mitosis. In contrast, meiosis is a prolonged process that can span hours to days leading to delayed SAC activation and silencing (Musacchio and Salmon, 2007; Homer et al., 2009; Gui and Homer, 2012; McGuinness et al., 2009). This delayed SAC response in meiosis may allow oocytes more time to correct alignment errors and promote the production of high-quality gametes (Lane et al., 2012). In summary, while SAC proteins share conserved roles in mitosis and meiosis, key differences in SAC sensitivity, timing, and response exist.

## 4 SAC in embryogenesis

Embryogenesis is a dynamic intricate process that encapsulates a multitude of temporal and spatial changes across the entire embryo for proper differentiation and maturation (Zhai et al., 2022). It occurs in all species and has been studied extensively in model organisms such as roundworms, fruit flies, frogs, fish, mice, and humans, although the process varies depending on the species (D'Costa and Shepherd, 2009; Technau, 1987). Cell division plays a critical role in embryogenesis as the zygote begins as a unicellular organism which undergoes multiple rounds of mitosis to promote multicellularity (Cinalli et al., 2008). Faithful segregation of chromosomes is equally essential during mitosis as it is during meiosis where aneuploid oocytes can lead to an increase in embryonic lethality as described above (Pan and Li, 2019; Jia et al., 2015). Interestingly, some organisms such as the fruit fly *Drosophila melanogaster* can tolerate aneuploidy. The loss of a copy of chromosome 4 is not lethal to *Drosophila* embryos, but results in smaller body size compared to wild-type flies (Bridges, 1921). Similarly, unicellular organisms like the fungus *Batrachochytrium dendrobatidis* exhibit extensive aneuploidy, suggesting that tolerance to aneuploidy varies across different organisms (Rosenblum et al., 2013). In contrast, mammals generally display lower tolerance towards aneuploidy, such as mosaic aneuploidy, where some cells in the body have an abnormal number of chromosomes (Garcia-Castillo et al., 2008). In mouse embryos, mosaic aneuploidy promotes embryo lethality by E8.0 through a p53-independent apoptotic mechanism (Lightfoot et al., 2006). Therefore, proteins that ensure proper chromosomal segregation during mitosis are essential for successful embryogenesis.

Once the male and female germ cells have fertilized, this produces a unicellular zygote which undergoes several rounds of cleavage to reach an 8-cell stage followed by an apical-basal polarization that allows for the segregation of distinct cell lineages (Zhu et al., 2021). Asymmetric cell divisions and the process of cavitation give rise to the blastocyst which then hatches from the zona pellucida and implants itself in the uterine lining (Zhu et al., 2021; Dey et al., 2004). In humans, this preimplantation development prior to hatching occurs within 5 days post fertilization (Dey et al., 2004). The SAC is active during preimplantation development (Wei et al., 2011). For instance, simultaneous overexpression of three SAC proteins (Bub3, BubR1, and Mad2) in one-cell embryos leads to inhibition of the metaphase to anaphase transition (Wei et al., 2011). Co-localization studies further revealed that all three SAC proteins are localized at the kinetochores. Conversely, RNAi-mediated depletion of Bub3, BubR1, or Mad2 disrupts the SAC and leads to anaphase onset with no apparent metaphase stage (Wei et al., 2011). The bypass of the SAC is further evidenced by the ability of RNAi-depleted embryos to progress to the two-cell stage despite nocodazole treatment, which normally arrests control embryos at the one-cell stage. These data indicate that SAC proteins are necessary for mitotic arrest in embryos (Wei et al., 2011). Karyotyping of embryos depleted of these SAC factors show a heightened level of aneuploidy and an accumulation of micronuclei. When these embryos are implanted in the uterine horn of mice, their viability are similar to control embryos (Wei et al., 2011). However, a substantially higher percentage of SAC depleted embryos display delayed development as compared to control embryos, demonstrating that the SAC is critical for mitotic progression and preimplantation development during embryogenesis (Wei et al., 2011).

### 4.1 SAC response across various species during embryogenesis

SAC strength and response has been shown to vary between species which became apparent when assessing responses of 2-cell stage embryos from nine different species, including both chordates and non-chordates, to nocodazole treatment (Chenevert et al., 2020). The chordates (*Phallusia mammillata*) continue to carry out mitosis even in the presence of nocodazole as suggested by oscillations in phosphorylated-Histone H3 levels (Chenevert et al., 2020). Whereas nocodazole induces a significant delay in mitotic progression in all other species that were either echinoderms or mollusks which was shown to be mediated by the SAC kinase Mps1 (Chenevert et al., 2020). Further examination of chordate embryos using live microscopy showed no difference in the duration of mitosis between control and nocodazole treated embryos (Chenevert et al., 2020). Since the chordate group continue to progress through mitosis even in the presence of spindle disruption, it indicates that the SAC is not efficient or active during the early embryonic developmental phase in chordates. Similar results were also observed in other species, including the invertebrates *Ciona intestinalis* and *Branchiostoma lanceolatum* (Chenevert et al., 2020). It is plausible to hypothesize that SAC kinases are not expressed during the early embryonic development which leads to a lack of SAC activity or mitotic delay in response to nocodazole treatment. However, the SAC proteins Mad1, Mad2, Bub1, Bub3, and Mps1 are all detected at the mRNA level during and after fertilization in *Phallusia mammillata* (Chenevert et al., 2020). Interestingly, fluorescence imaging revealed that Mps1, Mad1, and Mad2 are not recruited to unattached kinetochores in *P. mammillata* 2-cell embryos treated with nocodazole, suggesting that SAC assembly at the kinetochore is prevented during early embryonic development in chordates (Chenevert et al., 2020). It is important to note that BubR1 was not assessed in this study, leaving its role in this process unclear.

There are various hypotheses that have been proposed to address the differential SAC activity between various species. The first hypothesis postulates that the SAC response is dependent on cell size (Galli and Morgan, 2016). Evidence for this comes from a study conducted in *Caenorhabditis elegans* where the duration of mitotic arrests following nocodazole treatment was longer after each embryonic cell division from the 2-cell stage to 8-cell stage which correlates with a decline in the cell volume (Galli and Morgan, 2016). Interestingly, there was also a strong correlation between time from nuclear envelope breakdown to nuclear envelope reformation and cell size (Galli and Morgan, 2016). As the cell volume reduced with each consecutive division, SAC mediated mitotic delay became longer. Depletion of *ani-2*, an anillin homolog, produces *C. elegans* embryos of various sizes (Galli and Morgan, 2016). When *ani-2* was depleted alongside *zyg-1*, the Plk4 homolog that causes monopolar spindles, mitotic delays were longer in smaller size embryos further supporting the hypothesis that cell size may influence SAC activity. Along these same lines, since the ratio of unattached kinetochores to cytoplasmic volume increases as cell size decreases, it is possible that this kinetochore-to-cytoplasm ratio determines the strength of the SAC response (Galli and Morgan, 2016). In *C. elegans* heterozygous for rec-8(ok978), which produces triploid embryos with 50% more kinetochores than wild-type diploid embryos, the SAC signal was found to be stronger than in control diploid embryos of the same size. These results suggest that the kinetochore-to-cytoplasm ratio may influence the SAC response strength. However, this hypothesis is not supported by observations in other species (Vazquez-Diez et al., 2019). Live imaging studies of mouse embryos in the presence of nocodazole showed that the SAC was more efficient in the much larger 2-cell stage blastocyst than in smaller 4- or 8-cell stage morulae. Furthermore, when 40% of cytoplasm was removed to alter cell volume, nocodazole prolonged mitotic arrest in the 2-cell stage but did not extend the arrest in 4-cell stage (Vazquez-Diez et al., 2019). Similarly, this lack of correlation is evident in studies where SAC response and cell size were compared between species that produce embryos of various sizes. For instance, the blastomere of *P. mammilata* is 130 µm while a blastomere of *Drosophila* is 500 µm. Upon nocodazole treatment, the latter delays mitotic progression suggesting an efficient SAC response while the former fails to do so even with its smaller size (Chenevert et al., 2020). To rationalize these contradicting observations, it was proposed that the spindle architectural features should be considered along with cell size to explain mitotic timing and SAC efficiency (Bloomfield et al., 2020). Cytokinesis failure was induced in human diploid colon cancer DLD-1 cells to produce tetraploid cells of both small and large sizes. Smaller size 4N clones had longer mitotic arrest although the arrest time varied between the smaller size clones indicating that cell size itself may not be the primary factor (Bloomfield et al., 2020). Furthermore, the spindle pole size, spindle height, and spindle microtubule density were considered alongside cell size with results showing that all these factors influence SAC timing to various extents and thus may together control SAC efficiency (Bloomfield et al., 2020).

Another hypothesis that has been recently postulated asserts that the difference between SAC acquisition and SAC modulation is what influences SAC efficiency (Roca et al., 2023). Embryos from *P. mammillata*, which show weak SAC activity in the presence of nocodazole, exhibit similar mitotic duration up until the 7th cell cycle between control and nocodazole treated embryos (Roca et al., 2023). However, nocodazole treatment induced a prolonged mitotic delay from the 8th cycle onward suggesting that the SAC initiates at the 8th cycle (Roca et al., 2023). A dominant negative Mad2 hampered this SAC initiation at the 8th cycle while it had no effect prior to the 8^th^ cycle. While Mad1 expression is similar throughout development, immunofluorescence staining revealed that it does not accumulate on the mitotic chromosomes during the 4- to 32-cell embryos (Roca et al., 2023). However, Mad1 starts accumulating on the mitotic chromosomes during the 128- and 256-cell stage (Roca et al., 2023). This suggests that proper localization of SAC factors may define SAC proficiency in these embryos indicating that there may be an initial SAC-deficient phase followed by a later SAC-proficient phase during early embryonic development (Roca et al., 2023). The evolutionary benefit, if any, of the lack of SAC activity during early cell divisions remains to be determined.

Since embryogenesis leads to the development of various tissues, it is plausible that SAC efficiency could be influenced by different cell fates (Gerhold et al., 2018). This is supported by the observation that the anterior and posterior ventral ectodermal cells of *P. mammillata* embryos vary in their SAC strength (Roca et al., 2023). Moreover, cells within these two different regions are of the same size suggesting that the differences in SAC strength could be due to the different cell fates rather than cell size (Roca et al., 2023). The anterior cells can be induced to acquire posterior cell like identity through the inhibition of glycogen synthase kinase-3 (GSK3) (Feinberg et al., 2019). Interestingly, posterization of the anterior cells by GSK3 inhibition leads to a significant reduction in SAC efficiency in the presence of nocodazole (Roca et al., 2023). Similarly, posterior cells can be induced to undergo anterior fate by injecting the transcription factor Ci-FoxA-a (Lamy et al., 2006). Upon nocodazole treatment, the mitotic delay in Ci-FoxA-a injected posterior cells is 2.4-fold longer, further supporting the notion that cell fate may play a crucial part in regulating SAC strength (Roca et al., 2023). The relationship between cell fate and SAC efficiency has also been observed in *C. elegans* where germline fated cells display longer mitotic delays compared to somatic fated cells when spindle formation is perturbed in blastomeres with nocodazole (Gerhold et al., 2018). When germline and somatic cells of comparable sizes are treated with nocodazole, the germline cells still display a significantly longer mitotic delay suggesting that germline fated cells have a stronger SAC response (Gerhold et al., 2018). While the contribution of cell size cannot be completely eliminated in this model organism since the SAC strength increases as the blastomere undergoes cleavage and the cell size decreases, cell fate may have a greater role in governing SAC strength (Gerhold et al., 2018).

The SAC response might also be influenced by whether cells are undergoing constant division, as seen in rapidly dividing cells during early embryogenesis, or whether they are in a differentiated state with limited or no division. In early embryogenesis, rapidly dividing cells exhibit a more error-prone and less robust SAC response (Horakova et al., 2024). Potential reasons for this could be the rapid pace of division and incomplete checkpoint maturation. Early embryonic cells, particularly those undergoing cleavage divisions, lack cell cycle phases associated with interphase such as G1 and G2 that provide checkpoints to monitor and rectify DNA damage (Kumar et al., 2015). These cells undergo rapid cycling, often without sufficient time for quality control, leading to a higher incidence of aneuploidy (Horakova et al., 2024). For instance, aneuploidy rates are extremely high in oocytes and embryos compared to somatic cells (Vazquez-Diez and FitzHarris, 2018; Horakova et al., 2024). The cell cycle phases also vary significantly in *Xenopus*, *Drosophila*, and *C. elegans* embryos (Masui and Wang, 1998; Foe and Alberts, 1983; Edgar and McGhee, 1988). The durations of these phases lengthen as development progresses due to the acquisition of the G1 and G2 phases. Since the SAC operates during the mitotic phase, determining the duration of mitosis during development is of particular interest. A study on *Helobdella triserialis* embryos suggests that the duration of the mitotic phase remains constant during development, indicating that the SAC has sufficient time to function despite changes in other cell cycle phases (Bissen and Weisblat, 1989). In contrast, differentiated cells such as neurons do not undergo cell division, which might suggest that the SAC is inactive in these cells (Aranda-Anzaldo and Dent, 2017). However, SAC proteins such as Bub1 and Mad2 are present in neurons and influence microtubule dynamics suggesting that SAC proteins may functions beyond mitosis (Hertzler et al., 2020; Zhao et al., 2019; Cheerambathur et al., 2019). More research is needed to elucidate the roles of SAC proteins in embryonic versus differentiated cells and their potential non-mitotic functions in differentiated tissues.

Another intriguing perspective on SAC response is through the lens of evolution. Across eukaryotes, many species have retained SAC proteins and their conserved functions, such as *Saccharomyces cerevisiae* and *Homo sapiens* (Rudner and Murray, 1996). However, there are notable exceptions, including *Trypanosoma brucei*, which lacks a BubR1/Mad3 homolog, and *Giardia intestinalis*, which has lost the APC/C at some point in its evolutionary history (Akiyoshi and Gull, 2013; Gourguechon et al., 2013; van Hooff et al., 2017). In *T. brucei*, spindle assembly perturbation does not trigger a SAC response, as the cells proceed to cytokinesis despite the disruption, suggesting that the absence of key SAC components compromises SAC proficiency (Ploubidou et al., 1999; Akiyoshi and Gull, 2013). Interestingly, while such observations suggest a correlation between the presence of SAC proteins and SAC strength, there are exceptions. For example, the flatworm *Schmidtea mediterranea*, which lacks Mad2, BubR1/Mad3, and Bub1, still exhibits a delay in G2/M progression in response to nocodazole, implying the existence of alternative mechanisms to ensure chromosomal fidelity (Grohme et al., 2018). In addition, species such as *G. intestinalis* rely on Cyclin B degradation for cell cycle progression; however, unlike most organisms, the degradation of Cyclin B in *G. intestinalis* is not mediated by the APC/C. Instead, degradation is regulated through phosphorylation by unidentified upstream mechanisms, as *G. intestinalis* lacks the APC/C machinery (Gourguechon et al., 2013). Interestingly, ubiquitin ligases beyond APC/C have been identified as regulators of Cyclin B1 degradation, including CRL2^ZYG-11^ (Balachandran et al., 2016). This ligase, also found in *C. elegans* and human cells, operates redundantly with APC/C in targeting Cyclin B1 (Balachandran et al., 2016). Under normal conditions, when the APC/C is active, CRL2^ZYG-11^ inactivation has minimal effects. However, in the absence of APC/C activity, CRL2^ZYG-11^ compensates by degrading Cyclin B1, facilitating normal progression through metaphase (Balachandran et al., 2016). Cyclin B1 is essential for SAC function, as it serves as a scaffold for Mad1, anchoring it to the kinetochore corona. This localization enables Mps1 to phosphorylate Mad1 on its C-terminus (Allan et al., 2020). Once proper kinetochore-microtubule attachments are formed, Cyclin B1 is degraded by the APC/C to allow anaphase progression (Allan et al., 2020). However, in scenarios of sustained mitotic arrest, such as during nocodazole treatment, cells may bypass the SAC and exit mitosis via a process called mitotic slippage (Brito and Rieder, 2006). The mechanisms behind mitotic slippage have been debated, with evidence pointing to a gradual weakening of SAC signaling due to the loss of Mad2 at kinetochores (Lok et al., 2020). The discovery of CRL2^ZYG-11^ as a redundant Cyclin B1 regulator provides further insight into this phenomenon. Notably, depletion of CRL2^ZYG-11^ significantly reduces mitotic slippage in human cells, even under nocodazole treatment (Balachandran et al., 2016). Future studies exploring whether other species possess mechanisms beyond APC/C to degrade Cyclin B could shed light on the evolutionary diversity of SAC regulation and its implications for SAC proficiency.

The evolutionary origins of SAC components are particularly intriguing, as their presence across diverse eukaryotic species suggests that they likely originated in a common ancestor or the last eukaryotic common ancestor (LECA) (Kops et al., 2020). A recent review provides a comprehensive overview of the evolutionary branches emanating from the LECA and highlights how SAC components have diverged across eukaryotic lineages (Kops et al., 2020). Understanding the diversity of SAC components and their structural domains across species could shed light on the broad spectrum of SAC proficiency observed today. Such studies could offer valuable insights into the evolutionary adaptations that shape mitotic regulation across species.

Overall, the efficiency of the SAC during development is dictated by various factors whose level of contribution varies among species. It has also been speculated that SAC silencing is a by-product of changes that occur in reproductive regulation (Chenevert et al., 2020). It remains to be determined what exactly causes SAC function in the initial stages of embryonic development and what are the evolutionary benefits of the varied SAC activity in different species during early embryogenesis (Table 1).

**TABLE 1:** Relative SAC proficiency across different phyla and species.

Phylum	Species	SAC Competency	Literature
Chordates	*Phallusia mammillata*	-	Chenevert et al. (2020)
	*Ciona intestinalis*	-	Chenevert et al. (2020)
	*Branchiostoma lanceolatum*	-	Chenevert et al. (2020)
	*Xenopus laevis*	-	Gerhart et al. (1984)
	*Danio rerio*	-	Ikegami et al. (1997)
Nematodes	*Caenorhabditis elegans*	+	Encalada et al. (2005)
Arthropods	*Drosophila melanogaster*	+	Perez-Mongiovi et al. (2005)
Mollusks	*Mytilus galloprovincialis*	+	Milani et al. (1990)
	*Spisula solidissima*	+	Evans et al. (1983)
Cnidarians	*Clytia hemisphaerica*	+	Chenevert et al. (2020)
Echinoderms	*Hacelia attenuata*	+	Chenevert et al. (2020)
	*Paracentrotus lividus*	+	Chenevert et al. (2020)
	*Arbacia lixula*	+	Chenevert et al. (2020)

## 5 SAC proteins in gametogenesis and embryogenesis

The proteins involved in the SAC are broadly categorized into two groups: sensor and signal transducer proteins. Sensor proteins consist of Bub1, Mad1, and Mps1 while signal transducer proteins consist of the MCC proteins BubR1, Bub3, Mad2, and Cdc20 (Peters, 2006). The remainder of this review will discuss each of these SAC proteins and their role in gametogenesis and embryogenesis.

### 5.1 Bub1

Bub1 (budding uninhibited by benzimidazoles 1) is a serine/threonine kinase that is recruited to unattached kinetochores where it plays a crucial role in the assembly of the signal transducers (Kim and Gartner, 2021). Bub1 is recruited to the kinetochore by Bub3, along with BubR1. Both Bub1 and BubR1 also bind to the KI1 and KI2 motifs of Knl1, respectively (Kiyomitsu et al., 2007; Kiyomitsu et al., 2011; Krenn et al., 2012). Bub1 is one of the first proteins that arrives at unattached kinetochores where it activates the SAC by recruiting the Mad1-Mad2 complex (Kim and Gartner, 2021; Tang et al., 2004a). Therefore, Bub1 is vital for proper SAC function. The importance of Bub1 in embryogenesis and tissue homeostasis has been highlighted through the use of *Bub1* knockout mouse models (Perera et al., 2007). Homozygous *Bub1* null mice are embryonic lethal suggesting that Bub1 is essential for embryogenesis (Perera et al., 2007). *Bub1* null embryos die between E3.5 and E8.5 (Perera et al., 2007). Mouse embryonic fibroblasts with inactivated Bub1 display reduced proliferation, improper chromosome alignment, and aberrant mitosis, suggesting that Bub1 is essential for maintaining proper mitosis in developing embryos (Perera et al., 2007). E7.5 Bub1 deficient embryos also have reduced phosphorylated-histone H3 positive cells and mature spermatids in their seminiferous tubules (Perera et al., 2007). Deletion of Bub1 in post implantation embryos at E10.5 also leads to an arrested development within 48 h which indicates that Bub1 is essential to organogenesis (Perera et al., 2007; Tilston et al., 2009). Histological analysis of these embryos revealed an extensive hemorrhaging within the brain and the body cavity (Tilston et al., 2009). At a cellular level, Bub1 deleted embryos have a heightened level of apoptosis and a reduction in the number of miotic cells as demonstrated by reduced phosphorylated-histone H3 positive cells (Tilston et al., 2009). Overall, Bub1 is indispensable for normal embryogenesis.

In humans, studies of spontaneous miscarriages carried out in patients from Chongqing Medical University Affiliated Hospitals showed that almost half of embryos had reduced levels of Bub1 and aneuploidy (Shi et al., 2011). RNAi-mediated depletion of Bub1 in primary embryonic villus cells suppresses cell proliferation and increases aneuploidy (Shi et al., 2011). Biallelic germline mutations in *Bub1* that leads to a reduction in the total protein level and kinase activity have been identified in two patients (Carvalhal et al., 2022). These patients suffer from microcephaly, growth retardation, and cardiovascular defects, and impaired mitotic fidelity (Carvalhal et al., 2022). Overall, these studies suggest that Bub1 is essential for both embryogenesis and organogenesis.

### 5.2 Mad1 and Mad2

Mad1 is an adaptor protein that plays crucial role in the assembly of the MCC by recruiting Mad2 (Devigne and Bhalla, 2021). Mad2 is in an inactive (or open conformation) as it is recruited to the kinetochore (Musacchio, 2015a; Luo et al., 2018). Once it binds to Cdc20, Mad2 changes its conformation to its closed, active, conformation. This complex then functions with other SAC proteins to inhibit the APC/C (Luo et al., 2018). Mad1 knockout embryos do not survive embryogenesis while overexpression of Mad1 correlates with an increased frequency of early-lethality related to aneuploidy (Iwanaga et al., 2007; Zhao et al., 2021). This suggests that Mad1 expression needs to be finely tuned for effective SAC function during embryogenesis. Mad1 heterozygous knockout animals show a higher incidence of tumors such as melanoma, rhabdomyosarcoma, and osteosarcoma along with aneuploidy (Iwanaga et al., 2007). Factors that regulate Mad1 levels include miR-125b that binds to the 3′UTR region of *Mad1* and negatively regulates Mad1 expression levels (Zhao et al., 2021). Aneuploid embryos that undergo miscarriages have higher expression of Mad1 and a downregulation of miR-125b, suggesting that upstream regulators of Mad1 are essential for effective SAC response and prevention of aneuploidy (Zhao et al., 2021). Interestingly, the same study noted a decrease in expression of Bub3 protein which is another essential player of the SAC (Zhao et al., 2021).

*Mad2* knockout mice were utilized to study its role during embryogenesis (Dobles et al., 2000). When *Mad2* heterozygous mice were mated, no *Mad2* homozygous null pups were born suggesting that Mad2 is embryonic lethal and essential for embryogenesis (Dobles et al., 2000). Histological analysis of E7.5 *Mad2* null embryos revealed that they were smaller in size and had abnormal gross morphology compared to wild-type embryos (Dobles et al., 2000). TUNEL staining indicated that these embryos show enhanced apoptosis between E6.5-E7.5. They also displayed aberrant mitosis as demonstrated by reduced mitotic cells and a higher number of cells with abnormal anaphases (Dobles et al., 2000). *Mad2* null E5.5 embryos cultured *in vitro* also failed to arrest in mitosis in response to nocodazole treatment (Dobles et al., 2000). Overall, Mad2 is essential for embryonic viability and is required for proper chromosomal segregation in mice (Dobles et al., 2000). In humans, similar to Bub1, reduced levels of Mad2 are observed in cases of spontaneous miscarriages (Shi et al., 2011). Depleting Mad2 in embryonic villus cells leads to a higher percentage of cells with abnormal chromosomes and reduces cell proliferation (Shi et al., 2011).

*Mad2* overexpression has also been studied extensively in mice as Mad2 is overexpressed in many different tumor types such as Burkitt lymphomas and Plasmacytomas (Sotillo et al., 2007). Mouse embryonic fibroblasts (MEFs) that overexpress Mad2 show reduced proliferation and display tetraploidy with a higher incidence of apoptosis compared to control cells (Sotillo et al., 2007). Therefore, chromosomal instability is observed both when Mad2 is lost or overexpressed suggesting that its level must be precisely regulated for accurate cell division.

Since loss of Mad2 leads to embryonic lethality, conditional *Mad2* knockout mice were used to assess *Mad2* deletion in adult mice (Schukken et al., 2021). Systemic knockout of Mad2 in 9–10-week-old mice leads to atrophy in the intestines specifically in the jejunum and ileum, coinciding with an increase in apoptosis and mitotic abnormalities such as lagging chromosomes and anaphase bridges (Schukken et al., 2021). Interestingly, other organs including the lung, liver, and kidneys do not display any phenotype in response to systemic loss of Mad2 (Schukken et al., 2021). This suggests that various cell and tissue types respond differently to loss of SAC proteins, potentially due to differences in the frequency or rate of cell division in different organs. Tissues with higher rates of cell turnover, such as the intestine, may be more susceptible to mitotic defects caused by the loss of SAC proteins such as Mad2, while other organs with relatively slower or less frequent cell division may tolerate SAC protein loss. Therefore, Mad2 expression is crucial post embryogenesis especially in tissues that divide rapidly.

The interaction of Mad1 and Mad2 is also essential in gametogenesis specifically during synapsis which is the crossover between homologous chromosomes (Devigne and Bhalla, 2021). For instance, mutations that prevent Mad1:Mad2 interaction delays synapsis formation and abolishes the synapsis checkpoint in *C. elegans* (Devigne and Bhalla, 2021). Similarly, vertebrate oocytes require Mad1 and Mad2 for metaphase arrest during meiosis II (Tunquist et al., 2003). Overall, Mad1 and Mad2 are essential proteins whose precise regulation is critical for maintaining chromosomal stability during gametogenesis and embryogenesis. Their roles in the spindle assembly checkpoint are vital, with dysregulation leading to deleterious consequences, including aneuploidy and embryonic lethality.

### 5.3 Mps1

The kinase Mps1 is responsible for the initiation of the SAC and promoting MCC assembly (Lara-Gonzalez et al., 2021a; Fischer et al., 2021; Ji et al., 2017). Through its phosphorylation of Knl1, it provides a docking site for the SAC proteins at unattached kinetochores to successfully inhibit the activation of the APC/C (Ji et al., 2017). Mps1 activity is essential in both oogenesis and spermatogenesis. Mps1 deletion in *Drosophila* causes chromosomal segregation errors (Gilliland et al., 2007). Similarly, oocyte specific deletion of Mps1 impairs the segregation of chromosome in oocytes during meiosis I and negatively impacts the fertility of mice. In male mice, germ cell specific deletion of Mps1 depletes spermatocytes and causes infertility suggesting that Mps1 is essential for spermatogenesis (Fang et al., 2021). Mps1 is also crucial in embryogenesis. For instance, inhibition of Mps1 during the preimplantation stage of mouse embryos significantly disturbs the development of embryos from the 2-cell stage to the blastocyst stage (Ju et al., 2021). These embryos display misaligned chromosomes and an absence of kinetochore-microtubule attachment along with DNA damage and an increase in oxidative stress followed by heightened apoptosis (Ju et al., 2021). Overall, Mps1 is crucial for gametogenesis and embryogenesis which underscores the importance of Mps1 in ensuring accurate cell division and chromosomal stability during these processes.

### 5.4 BubR1

Budding uninhibited by benzimidazole-related 1 (BubR1), encoded by the *Bub1b* gene, is a component of the MCC along with Bub3, Mad2, and Cdc20 (Bolanos-Garcia and Blundell, 2011). The MCC blocks the activation of the APC/C complex thereby preventing ubiquitination and degradation of key anaphase onset substrates such as Securin and Cyclin B1 (Sudakin et al., 2001; Reddy et al., 2007). Homozygous BubR1 knockout embryos fail to survive beyond E6.5 *in utero* (Wang et al., 2004). Blastocysts from wild-type and heterozygous knockout mice are able to hatch from the zona pellucida, attach to culture plates, and proliferate by E6.5 (Wang et al., 2004). BubR1 knockout embryos also display slower proliferation and atrophy by E6.5 with heightened levels of cells undergoing apoptosis as revealed by TUNEL staining (Wang et al., 2004) Adult *BubR1* ^*+/−*^ mice also show splenomegaly and extramedullary megakaryopoiesis (Wang et al., 2004). Aneuploidy was not examined in the knockout embryos although it can be speculated that the higher level of cell death may be due to chromosomal instability in the BubR1 knockout embryos (Wang et al., 2004). BubR1 also plays a crucial role in regulating kinetochore-microtubule attachments. Cells depleted of BubR1 fail to form stable microtubule attachments to kinetochores (Lampson and Kapoor, 2005). Similarly, depletion of Bub1 also disrupts these attachments, suggesting that the deletion of either Bub1 or BubR1 contributes to aneuploidy not only by impairing SAC function but also by causing unstable kinetochore-microtubule interactions (Lampson and Kapoor, 2005).

Mammalian oocytes undergo two phases of meiosis to produce secondary oocytes that can fertilize with a mature spermatozoon (MacLennan et al., 2015). Oocytes depleted of BubR1 cannot carry out prophase I arrest during meiosis I and undergo germinal vesicle breakdown (MacLennan et al., 2015). BubR1 depletion in oocytes leads to a significant reduction in CDH1, a co-activator of APC/C, which is required for APC/C led arrest during prophase I (Homer et al., 2009). Interestingly, these BubR1 depleted oocytes become arrested before the completion of meiosis I and fail to produce polar bodies (Homer et al., 2009). In contrast, another study demonstrated that BubR1 depleted oocytes do not undergo cell cycle arrest even in the presence of nocodazole and successfully produce polar bodies, which might be attributed to various degrees of BubR1 depletion achieved in these contradictory studies (Wei et al., 2010). Using mouse oocytes completely devoid of BubR1 revealed that BubR1 is not required for prophase I arrest during meiosis (Touati et al., 2015). As a result, BubR1 knockout oocytes undergo germinal vesicle breakdown and readily carry out meiosis I in an accelerated manner suggesting that SAC function is impaired in the absence of BubR1 (Touati et al., 2015). Kinetochore-microtubule interactions that are resistant to depolymerization when exposed to cold temperatures are absent in BubR1 deleted oocytes indicating that BubR1 loss reduces stability of spindle fibers in oocytes (Touati et al., 2015). Furthermore, BubR1 depleted oocytes that complete meiosis I and II are found to be aneuploid (Touati et al., 2015). Overall, these studies indicate that BubR1 is essential for regulating the timing of oogenesis and the production of healthy oocytes.

To study the role of BubR1 in adult mice, BubR1 hypomorphic mice expressing approximately 10% of BubR1 protein found in wild type mice were engineered (Baker et al., 2004). BubR1 hypomorphic mice display severe phenotypes including aneuploidy and premature aging phenotypes such as growth retardation, cataracts, sarcopenia, and lordokyphosis (Baker et al., 2004). BubR1 hypomorphic mice also exhibit cardiac anomalies, including prolonged QT syndrome, decreased tolerance to cardiac stress following injection with the β-adrenergic agonist isoproterenol, and a tendency to die in a manner reminiscent of sudden cardiac death (Baker et al., 2004; Baker et al., 2013; North et al., 2014). Mutation of BubR1 in humans leads to Mosaic Variegated Aneuploidy (MVA) syndrome, which is characterized by increased incidences of tumorigenesis, and progeroid traits such as shortened lifespan, cataracts, congenital heart defects, and facial dysmorphism (Suijkerbuijk et al., 2010). Overall, these studies indicate that BubR1 is essential for oogenesis, embryogenesis, and maintenance of tissue/organs in adulthood.

### 5.5 Bub3 and Rae1

Budding uninhibited by benomyl (Bub3) is a component of the MCC complex along with BubR1, Cdc20, and Mad2. Bub3 is a crucial component of the SAC since it binds to phosphorylated MELT repeats on the outer kinetochore subunit Knl1 that leads to the recruitment of Bub1 and BubR1 to kinetochores (Kiyomitsu et al., 2007; Krenn et al., 2012; Yamagishi et al., 2012; Primorac et al., 2013). Bub3 binds to Bub1 and BubR1 through their GLEBS motifs (Taylor et al., 1998). Mice that harbor *Bub3* null alleles were generated, and similar to other MCC components, they are embryonic lethal and die between E6.5-E7.5 (Kalitsis et al., 2000). Blastocysts obtained at day E3.5 were cultured *in vitro* and examined for morphological abnormalities (Kalitsis et al., 2000). While control embryos rapidly divided and increased their inner cell mass, *Bub3* null embryos remained smaller in size and degenerated, suggesting they are alive at E3.5 but eventually die (Kalitsis et al., 2000). These embryos were also found to be resorbed prior to E8.5 *in utero* (Kalitsis et al., 2000). Furthermore, mitotic abnormalities are present in the *Bub3* null embryos starting at E3.5 and as the embryos progress, they accumulate more mitotic abnormalities including the formation of micronuclei, nuclear bridging, and abnormal nuclei (Kalitsis et al., 2000). These data suggest that *Bub3* is required for proper early embryogenesis.

Bub3 shares extensive sequence homology with Rae1 (Babu et al., 2003). Rae1, although not a well-characterized SAC component, has been implicated in processes that influence mitotic progression and checkpoint functions (Martinez-Exposito et al., 1999; Taylor et al., 1998; Babu et al., 2003). Rae1 was originally found to be involved in mRNA export during interphase where Rae1 binds to the GLEBS motif of the nucleoporin Nup98 (Brown et al., 1995; Wang et al., 2001). Similar to Bub3, Rae1 also binds to the GLEBS domain in both Bub1 and BubR1 to regulate the SAC complex (Wang et al., 2001). *Rae1* null embryos die by E8.5 (Babu et al., 2003). At E3.5, *Rae1* null embryos are morphologically indistinguishable from wild type and heterozygous littermates (Babu et al., 2003). Blastocysts harvested at E3.5 continue to hatch from the zona pellucida, but *Rae1* null embryos fail to divide their inner cell mass and degenerate by E8.5 (Babu et al., 2003). Interestingly, *Rae1* null embryos are able to carry out nuclear mRNA export suggesting that failure to export nuclear mRNA due to loss of Rae1 is not responsible for the observed embryonic lethality (Babu et al., 2003). When MEFs isolated from heterozygous *Rae1* null embryos were challenged with nocodazole to examine the effect of Rae1 loss on the response to spindle damage, Rae1 haploinsufficient cells were unable to arrest in mitosis and showed significantly higher levels of chromosomal missegregation (Babu et al., 2003). Similarly, Bub3 haploinsufficiency also leads to failure in mitotic checkpoint arrest and errors in chromosome segregation when treated with nocodazole (Babu et al., 2003). When Rae1 and Bub3 haploinsufficiency are combined, an increase in aneuploidy both *in vitro* and *in vivo* was observed (Babu et al., 2003). Interestingly, *Rae1* overexpression reduces the mitotic checkpoint defects observed in *Bub3* haploinsufficient MEFs suggesting that Rae1 can compensate for the reduction of Bub3 with respect to SAC function (Babu et al., 2003). Furthermore, combined haploinsufficiency of Rae1 and Bub3 leads to increased tumorigenesis in mice due to an increase in chromosomal instability (Babu et al., 2003). Taken together, these findings suggest that both Bub3 and Rae1 are essential for SAC function during embryogenesis.

### 5.6 Cdc20

Cdc20 is another member of the MCC complex and also serves as a substrate recognition subunit of the E3 ubiquitin ligase APC/C promoting ubiquitination of its substrates (Qiao et al., 2016). The phosphorylation of the APC/C by CDK1 promotes the binding of Cdc20 to the APC/C (Golan et al., 2002; Kramer et al., 2000). The formation of APC/C-Cdc20 complex allows for the recognition of APC/C substrates important for the metaphase-to-anaphase transition. The WD-40 domain in Cdc20 recognizes degron motifs in substrate proteins allowing for their recruitment to the APC/C and subsequent ubiquitination (Kraft et al., 2005; Glotzer et al., 1991). Cdc20 is essential for successful embryogenesis, as its deletion in mouse embryos leads to embryonic lethality. Analysis of *Cdc20* deleted embryos showed that these embryos arrest at E3.5 at the two-cell stage (Li et al., 2007). When E1.5 embryos were harvested and cultured *in vitro*, the *Cdc20* null embryos failed to progress from the 2-cell stage to the 4-cell stage (Li et al., 2007). Cdc20 bound to the APC/C mediates the ubiquitination of Securin and Cyclin B1 followed by the activation of Separase and the onset of anaphase (Li et al., 2007). *Cdc20* null embryos at E1.5 demonstrate a high level of Cyclin B1 and Securin suggesting that *Cdc20* null embryos fail to transition to anaphase causing metaphase arrest (Li et al., 2007). Meanwhile, Cdc20 and Securin double mutants were unable to maintain the metaphase arrest suggesting that Securin is important for the metaphase arrest caused by Cdc20 deletion (Li et al., 2007). Homozygous mutant embryos that have Cdc20 lacking the Mad2 binding domain are also not viable and die before E14.5 (Li et al., 2009a). Morphologically, the mutant embryos are smaller and suffer from massive apoptosis (Li et al., 2009a). Upon nocodazole treatment, mutant cells fail to undergo mitotic arrest (Li et al., 2009a). Moreover, reduction in Cdc20 activity, using heterozygous *Cdc20* mice, leads to chromosomal instability and an increase in tumorigenesis (Li et al., 2009a).

In humans, mutations in Cdc20 were identified in a cohort of infertile individuals who displayed oocyte maturation arrest, fertilization failure, and early embryonic arrest (Zhao et al., 2020). These mutations included a missense mutation c.965G > A (p. Arg322Gln) and nonsense or frameshift mutations (c.544C > T [p.Arg182*], c.813_814ins AGTG [p.Gly272Serfs*24], and c.1176_1179del TCTG [p.Cys392*]) (Zhao et al., 2020). The missense mutation (p.Arg322Gln) showed normal kinetochore localization of Cdc20 in oocytes, similar to the wild type protein (Zhao et al., 2020). However, the frameshift and nonsense mutations (p.Arg182*, p.Gly272Serfs*24, and p.Cys392*) caused defective kinetochore localization, leading to a loss-of-function phenotype (Zhao et al., 2020). In transfected chinese hamster ovary (CHO) cells, these mutations resulted in reduced Cdc20 protein levels or truncated Cdc20 proteins. Additionally, these mutations caused a decrease in Cyclin B1 degradation, which is a critical function of Cdc20 during the metaphase-to-anaphase transition (Zhao et al., 2020). The mutations also resulted in reduced mRNA expression of Cdc20 further indicating that these mutations lead to unstable Cdc20 protein and degraded mRNA. Injection of *Cdc20* cRNA rescued these phenotypes in the mutated oocytes indicating that Cdc20 is essential for proper oocyte development and fertility in humans (Zhao et al., 2020). Furthermore, treatment with the Cdc20 inhibitor Apcin interfered with embryo implantation in mice (Guo et al., 2020). Cdc20 expression reaches its peak during the estrus phase of menstrual cycle in mouse endometrial tissue and during the mid-secretory phase in human endometrial tissue (Guo et al., 2020). Apcin treatment reduced the proliferation and adhesion rate of human uterine epithelial cells lines HEC-1A and RL95-2, and significantly reduced the number of implanted embryos as compared to control groups (Guo et al., 2020). Taken together, Cdc20 appears to be essential for embryogenesis, embryo implantation, and fertility in mammals.

## 6 Conclusion

In summary, SAC proteins play a vital role in gametogenesis and embryogenesis. Knocking out most SAC proteins leads to embryonic lethality while inducible systemic knockout in adult animals negatively impacts the functions of various organs (Table 2). SAC function and efficiency seem to vary between species and are affected by different factors including cell fate, cell size, and kinetochore-to-cytoplasm ratio. The molecular basis and functional consequences of this species variation in SAC function should be studied in more depth to decipher what factors regulate SAC efficiency during embryogenesis. Moreover, cellular pathways that regulate the activity level of SAC is a substantial gap in knowledge that is yet to be explored in detail which is imperative since SAC response appears to undergo a period of silencing during the initial period of embryogenesis in some organisms. Such analysis may also shed light on the potential evolutionary benefit of regulating SAC activity in some species that holds potential for clinical therapy such as in aneuploidy and cancer that arise as a result of chromosomal instability.

**TABLE 2:** Lethality age and phenotypes displayed by various *in vivo* and *in vitro* models of SAC proteins.

SAC Protein	Model	Lethality Age	Phenotypes	Literature
Bub1	*BUB1* null, *BUB1* ^Δ/Δ^	E3.5- E8.5	- Hemorrhaging within the periphery of the embryo, the brain and body cavity - Increase in apoptosis - Reduced number of mitotic cells - Abnormal seminiferous tubules - Infertility in males	Tilston et al. (2009), Perera et al. (2007)
	*BUB1* ^*F/Δ* ^MEFs; *ERT-Cre*	NA	- Reduced viability and proliferation	Perera et al. (2007)
Mad1	*Mad1* ^*−/−*^	NA	- Embryonic lethal	Iwanaga et al. (2007)
	*Mad1* ^*−/+*^	NA	- Increase tumor incidence - Aneuploidy - Attenuated SAC response	Iwanaga et al. (2007)
Mad2	*Mad2* ^−/−^	E6.5–E7.5	- Increase in apoptosis	Dobles et al. (2000)
	*Mad2* ^−/−^ embryonic cells cultured *in vitro*	NA	- SAC deficient in response to nocodazole treatment - High rate of chromosomal missegregation	Dobles et al. (2000)
	*Mad2l* ^*fl/fl*^ *; Cre-ERT2*	2–3 weeks post knockout initiation	- Significant loss of weight - Rapid atrophy in jejunum/ileum - Increase in apoptosis cells - Mitotic abnormalities in intestine	Schukken et al. (2021)
Mps1	Mps1 specific inhibitor (Mps1-1N-1)	N/A	- Arrested progression from 2-cell stage to blastocysts - Kinetochore-microtubule attachment defects - DNA damage and oxidative stress in mouse embryos - Increase apoptosis and autophagy	Ju et al. (2021)
BubR1	*BUBR1* ^−/−^	E5.5-E7.5	- Increase in apoptosis - Splenomegaly - Extramedullary megakaryopoiesis	Wang et al. (2004)
	*BUBR1* ^−*/+*^ MEFs	N/A	- Increase in polyploidy - Defective SAC control	Wang et al. (2004)
	*Bub1b* ^*H/H*^	∼ 6 months	- Shortened lifespan - Cachectic dwarfism - Lordokyphosis - Cataracts - Impaired wound healing	Baker et al. (2004)
	*Bub1b* ^*H/H*^ MEFs	N/A	- Defects in Mitotic checkpoint - Heightened senescence	Baker et al. (2004)
Bub3	*Bub3* ^*−/−*^	E6.5-E7.5	- Incompetent SAC - Formation of micronuclei - Lagging chromosome - Irregular nuclear morphology	Kalitsis et al. (2000)
	*Bub3* ^*+/−*^ MEFs	N/A	- Reduced mitotic index - Increase in chromosomal instability	Babu et al. (2003)
Rae1	*Rae1* ^*−/−*^	E3.5-E8.5	- Embryonic lethal	Babu et al. (2003)
	*Rae1* ^*+/−*^ MEFs	N/A	- Defective mitotic checkpoint - Increased chromosomal instability	Babu et al. (2003)
Cdc20	*Cdc20* ^*gt/gt*^ null embryos	Two-cell stage	- Arrested in metaphase - High levels of Cyclin B1	Li et al. (2007)
	*Cdc20* ^*AAA/AAA*^	E12.5-E14.5	- Compromised SAC	Li et al. (2009a)
	*Cdc20* ^*AAA/AAA*^ MEFs	N/A	- Reduced mitotic index - Lower cell number - Aneuploidy - Higher incidences of spontaneous tumorigenesis in *Cdc20* ^*AAA/+*^ mice	Li et al. (2009a)
